# Quantitative analysis of ciliary beating in primary ciliary dyskinesia: a pilot study

**DOI:** 10.1186/1750-1172-7-78

**Published:** 2012-10-11

**Authors:** Jean-François Papon, Laurence Bassinet, Gwenaëlle Cariou-Patron, Francoise Zerah-Lancner, Anne-Marie Vojtek, Sylvain Blanchon, Bruno Crestani, Serge Amselem, Andre Coste, Bruno Housset, Estelle Escudier, Bruno Louis

**Affiliations:** 1 INSERM, U955, Equipe 13, Créteil 94000, France; 2 Universite Paris Est, Faculté de Médecine, Créteil 94000, France; 3 AP-HP, Hôpital H.-Mondor – A. Chenevier et Hopital intercommunal, service d’ORL et de chirurgie cervico-faciale, Creteil 94000, France; 4 INSERM, U933, Paris 75012, France; 5 CNRS, ERL 7240, Creteil 94000, France; 6 Hôpital intercommunal, service de pneumologie, Créteil 94000, France; 7 AP-HP, Hôpital H.-Mondor – A. Chenevier, service de physiologie et d’explorations fonctionnelles, Créteil 94000, France; 8 Hôpital intercommunal, service d’anatomo-pathologie (laboratoire de microscopie electronique), Créteil 94000, France; 9 AP-HP, Hôpital Bichat-Claude Bernard, service de pneumologie A, Centre de Compétence pour les maladies pulmonaires rares, Paris 75018, France; 10 Universite Paris Diderot, Paris 775018, France; 11 Universite Pierre et Marie Curie, Paris 75013, France; 12 AP-HP, Hôpital Armand-Trousseau, service de genetique et d’embryologie medicales, Paris 75012, France; 13 Consultation ORL, Hopital H. Mondor, 51, avenue du Marechal de Lattre de Tassigny, Creteil Cedex 94010, France

**Keywords:** Cilia, Electron microscopy, High-speed videomicroscopy, Kartagener syndrome, Nitric oxide

## Abstract

**Background:**

Primary ciliary dyskinesia (PCD) is a rare congenital respiratory disorder characterized by abnormal ciliary motility leading to chronic airway infections. Qualitative evaluation of ciliary beat pattern based on digital high-speed videomicroscopy analysis has been proposed in the diagnosis process of PCD. Although this evaluation is easy in typical cases, it becomes difficult when ciliary beating is partially maintained. We postulated that a quantitative analysis of beat pattern would improve PCD diagnosis. We compared quantitative parameters with the qualitative evaluation of ciliary beat pattern in patients in whom the diagnosis of PCD was confirmed or excluded.

**Methods:**

Nasal nitric oxide measurement, nasal brushings and biopsies were performed prospectively in 34 patients with suspected PCD. In combination with qualitative analysis, 12 quantitative parameters of ciliary beat pattern were determined on high-speed videomicroscopy recordings of beating ciliated edges. The combination of ciliary ultrastructural abnormalities on transmission electron microscopy analysis with low nasal nitric oxide levels was the “gold standard” used to establish the diagnosis of PCD.

**Results:**

This “gold standard” excluded PCD in 15 patients (non-PCD patients), confirmed PCD in 10 patients (PCD patients) and was inconclusive in 9 patients. Among the 12 parameters, the distance traveled by the cilium tip weighted by the percentage of beating ciliated edges presented 96% sensitivity and 95% specificity. Qualitative evaluation and quantitative analysis were concordant in non-PCD patients. In 9/10 PCD patients, quantitative analysis was concordant with the “gold standard”, while the qualitative evaluation was discordant with the “gold standard” in 3/10 cases. Among the patients with an inconclusive “gold standard”, the use of quantitative parameters supported PCD diagnosis in 4/9 patients (confirmed by the identification of disease-causing mutations in one patient) and PCD exclusion in 2/9 patients.

**Conclusions:**

When the beat pattern is normal or virtually immotile, the qualitative evaluation is adequate to study ciliary beating in patients suspected for PCD. However, when cilia are still beating but with moderate alterations (more than 40% of patients suspected for PCD), quantitative analysis is required to precise the diagnosis and can be proposed to select patients eligible for TEM.

## Background

Primary ciliary dyskinesia (PCD) is a rare congenital respiratory disorder with an estimated prevalence of 1:15–30,000 live births [1]. PCD is characterized by abnormal ciliary motility usually related to an ultrastructural defect. Ciliary dysfunction, affecting mucociliary clearance, leads to chronic airway infections characterized by bronchiectasis and chronic sinusitis, sometimes associated with *situs inversus* (Kartagener syndrome) and male infertility [2]. Although airway symptoms usually begin in early childhood, the diagnosis is sometimes delayed for several years [1,3-5]. However, it is of prime importance to recognize this disease early in order to start appropriate therapy of respiratory tract infections and minimize lung damage.

In the presence of a suggestive clinical presentation, the diagnosis of PCD is usually based on the detection of abnormal motility and ultrastructural defects found in most of the cilia [3]. However, the diagnosis of PCD is difficult in up to 30% of patients because transmission electron microscopy (TEM) analysis is either unfeasible or ultrastructure is normal or incompletely decipherable [6,7]. Nasal nitric oxide (NO_n_), known to be dramatically reduced (10-15% of normal) in most patients with confirmed PCD, has been proposed to improve diagnostic procedures [8-11]. However, NO_n_ is sometimes difficult to measure, especially in young children, and low NO_n_ values have been reported in other disorders with overlapping clinical features such as cystic fibrosis [10-12].

It has been recently demonstrated that slow motion analysis of ciliary beating using digital high-speed videomicroscopy is more sensitive and specific than ciliary beat frequency measurement alone for patient selection before TEM analysis of cilia [13]. Some specific ultrastructural ciliary defects observed in PCD are associated with a characteristic abnormal beat pattern [14]. However, in our clinical practice, although description of beat pattern is easy in typical cases as immotile cilia, it becomes highly subjective when ciliary beating is totally or partially maintained. Consequently, our assumption was that quantitative analysis of ciliary beating would improve identification of ciliary beat pattern abnormalities.

In the present study, we determined parameters allowing quantitative analysis of ciliary beating and compared the results with those provided by qualitative evaluation in patients in whom the diagnosis of PCD was confirmed or excluded. We also applied this analysis to patients with an inconclusive diagnosis.

## Methods

### Patients

TEM analysis of cilia, NO_n_ measurement and study of ciliary beat pattern using digital high-speed videomicroscopy were performed in 34 patients consecutively referred to our PCD diagnostic center. All patients were investigated because of chronic upper and/or lower respiratory tract infections, i.e., bronchitis and/or bronchiectasis and sinusitis, possibly related to PCD. Other pathologic conditions such as cystic fibrosis, α1-antitrypsin deficiency, or humoral immune defect had been previously excluded. Patient history was reviewed for respiratory diseases and tobacco addiction. The presence of bronchiectasis and *situs inversus* were assessed on high-resolution chest computed tomography (HRCT) scan in all patients. Patients with unfeasible TEM and/or NO_n_ measurement were excluded from the study. All investigations were performed in the absence of acute airway infection for at least 6 weeks. Informed consent was obtained from all patients, and this study was approved by the Ile de France Ethics Committee.

### TEM analysis of ciliary ultrastructure

Biopsies of ciliated epithelium were obtained from the inferior nasal turbinate and processed for electron microscopy as previously described [15]. Ciliary ultrastructural results were expressed as a percentage of abnormal cilia among the total number of cilia analyzed [16]. For each ciliary ultrastructural study, axonemal abnormalities were quantified, and the ultrastructural phenotype was defined by the main ultrastructural defect (involving the dynein arms or microtubules). Dynein arms were considered to be absent from axonemal sections when the structure was missing from at least five of the nine peripheral doublets.

### NO_n_ measurement and pulmonary function tests

NO_n_ was measured according to international guidelines [17] using a chemiluminescent nitric oxide analyzer (EVA4000, Series, Aix en Provence, France), as previously described [18]. Results were expressed as NO_n_ output (nl/min). The normal value of NO_n_ output is higher than 150 nl/min in healthy subjects [9] and NO_n_ <100 nl/min was considered to be the cutoff value to distinguish PCD from non-PCD individuals [8,10,11].

Spirometry measurements and flow-volume curves were obtained using a spirometer (MedGraphics, PF/DX 1085D, St. Paul, MN).

### Study of ciliary beating using digital high-speed videomicroscopy

Ciliated samples were obtained by brushing the middle part of the inferior turbinate with a 2 mm cytology brush (Laboratoires Gyneas, Goussainville, France). Cells were suspended in B1 BSA medium (Laboratoire CCD, Paris, France) and examined within three hours. All observations, at 37°C with an inverted microscope (Axiovert 200, Carl Zeiss S.A.S. Le Pecq France) using an oil immersion x100 objective, were performed within 20 min. Beating ciliated edges were recorded with a digital camera (PixeLINK A741, Ottawa, Canada) at a rate of 355 frames per second. Each movie was composed of 1,800 frames with a definition of 256 × 192 pixels. Pixel size was (0.13 × 0.13) μm^2^. An example of this video sequence is available on Additional file 1: Video 1. Twenty distinct areas containing intact undisrupted ciliated epithelial edges greater than 50 μm, devoid of mucus and beating in the plane of the camera were recorded. As recommended [19], isolated ciliated cells were excluded. The study of ciliary beating was performed with no knowledge of the patients’ data (clinical status, NO_n_ and TEM).

#### Qualitative evaluation of ciliary beat pattern

Types of beat pattern were inferred according to the description published by Chilvers et al. [14]. The percentage of each type of beat pattern (i.e. normal, virtually immotile, stiff or circular) was determined in each patient. Mean ciliary dyskinesia score was also inferred for each patient as described by Chilvers et al. [20]. Briefly, normal coordinated ciliary beating was scored as 0 and dyskinetic beating was scored from 1–3 depending on the extent of abnormal beating along the edge. A score ≥ 2 was described as the best predictor of PCD [13].

#### Quantitative analysis of ciliary beat pattern

**Figure 1:**
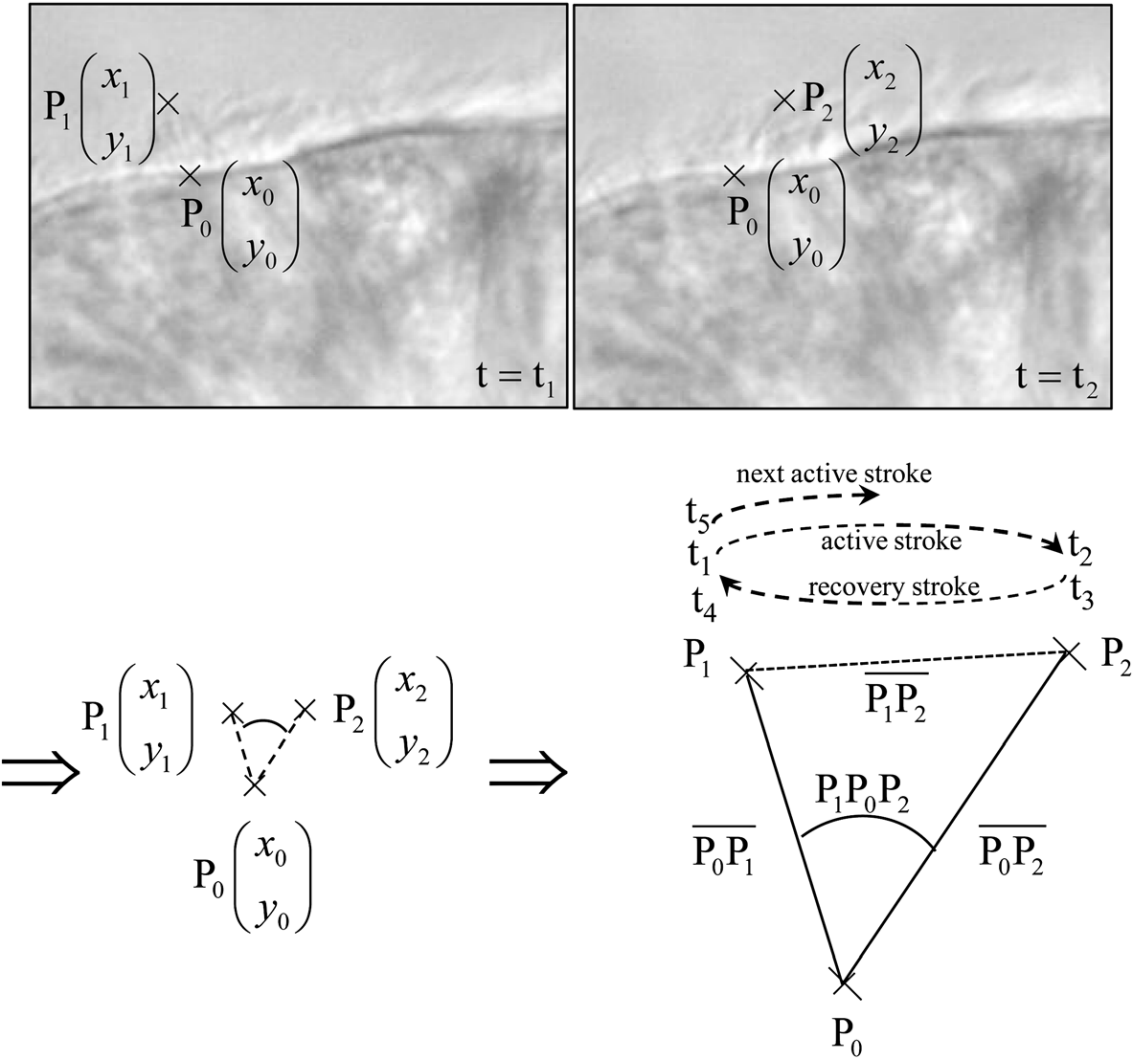
**Determination of digital high-speed videomicroscopy parameters of ciliary beat pattern of a cilium.** The three positions of the cilium (base of the cilium, P_0_, positions of the tip before the active and recovery strokes, P_1_ and P_2_ respectively) and the five time-points of beating (start and arrival of the active stroke, t_1_ and t_2_ respectively, start and arrival of the recovery stroke, t_3_ and t_4_ respectively, and start of the following cycle, t_5_) were measured. Twelve parameters were then determined from these measurements:. * The cilia length: $L_{c}\text{=max}\left({\bar{P_{0}P}}_{1},\bar{P_{0}P_{2}}\right)$ where $\bar{P_{i}P_{j}}$ designates the euclidean distance between the points P_i_ and P_j_. In cartesian coordinates $\bar{P_{i}P_{j}}=\sqrt{{\left(x_{i}-x_{j}\right)}^{2}+{\left(y_{i}-y_{j}\right)}^{2}}$ *The angle of beating, $\hat{{\;P}_{1}P_{0}P_{2}},$ is given by the law of cosines: $\hat{{\;P}_{1}P_{0}P_{2}}\text{=Arcos}\left(\frac{{{\bar{P_{0}P}}_{1}}^{2}+{\bar{P_{0}P_{2}}}^{2}-{\bar{P_{1}P_{2}}}^{2}}{2\cdot {\bar{P_{0}P}}_{1}\cdot \bar{P_{0}P_{2}}}\right)$ *The global frequency: $\mathrm{Fg=}\frac{1}{\left(t_{5}{-t}_{1}\right)}$ *The power stroke duration: Dp=(t_2_ - t_1_) *The recovery duration: Dr=(t_4_ - t_3_) *The pause after stroke: Ps=t_3_ - t_2_ *The pause after recovery: Pr=t_5_ - t_4_ *The total pause: Pt=Ps+Pr *The distance travelled per ${\text{second = L}}_{c}\cdot \hat{P_{1}P_{0}P_{2}}\cdot f_{g}\cdot 1\;\text{second}$ *The area swept $\text{per second =}\hat{P_{1}P_{0}P_{2}}/2\cdot L_{c}^{2}\cdot f_{g}\cdot 1\;\text{second}$ *The weighted distance travelled $\text{per second = \% of beating edges}\cdot L_{c}\cdot \hat{P_{1}P_{0}P_{2}}\cdot f_{g}\cdot 1\;\text{second}$ *The weighted area swept $\text{per second = \% of beating edges}\cdot \hat{P_{1}P_{0}P_{2}}/2\cdot L_{c}^{2}\cdot f_{g}\cdot 1\;\text{second.}$

In each patient, the percentage of beating ciliated edges was first determined. Briefly, in each of the 20 areas, an epithelial edge with a majority of cilia beating was scored 1; an edge with half of cilia beating was scored 0.5 and an edge with a minority of cilia beating was scored 0. The percentage of beating ciliated edges was defined as the sum of the scores divided by 20. Ten cilia able to be followed during a complete beating cycle (excluding cilia whose tip ran out of the focal plane) were then selected in distinct edges. Video sequences were played back frame by frame in order to determine three points characterizing the complete cycle of each cilium. These three points correspond to the position of the base of the cilium (P0) and the positions of the tip before the active and recovery strokes (P1 and P2, respectively) (see Figure 1). Five time-points were defined at the tip of the cilium corresponding to the steps of one beating cycle: start and arrival of the active stroke (t1 and t2, respectively), start and arrival of the recovery stroke (t3 and t4, respectively) and start of the following beating cycle (t5). These various measurements were used to determine 12 parameters. Global frequency, power stroke duration and recovery duration, the pauses after the active and recovery strokes, total pause, cilia length and beating angle were calculated. The distance travelled by the cilium per second (i.e., the length of the path travelled by the tip in one second) and the area swept per second (i.e., the area swept by the cilium in one second) were also calculated. These two last parameters were then weighted by the percentage of beating ciliated edges. The formula used to calculate these different parameters is shown in the legend to Figure 1.

### Statistical analysis

The set of results of ciliary ultrastructure and NO_n_ measurement was used as the “gold standard” for PCD diagnosis in order to compare the efficiency of qualitative and quantitative studies of ciliary beating in establishing the diagnosis of PCD. The PCD group was defined by a combination of > 90% abnormal cilia all sharing the same ultrastructural defect with NO_n_ < 100 nl/min. The non-PCD group was defined by a combination of <20% of abnormal cilia with NO_n_**≥** 100 nl/min and the inconclusive group was defined by discordant TEM and NO_n_ results.

Comparison of age, FEV_1_, FEV_1_/FVC (FEV1 is the forced expiratory volume in 1 second and FVC is forced vital capacity) and PaO_2_ between the three groups was performed with a statistical software package (Statistica v7) using the Kruskal-Wallis test (nonparametric one-way analysis of variance). The mean results of each digital high-speed videomicroscopy parameter were compared between the PCD and non-PCD groups using a Mann–Whitney *U* test (nonparametric test). A p value < 0.05 was considered significant. The estimated sensitivity and specificity of each parameter of the quantitative analysis were evaluated in the PCD and non-PCD groups, using ROC curve and area under curve (AUC). An AUC equal to 1 corresponded to 100% sensitivity and specificity, while a value of 0.5 corresponded to a sensitivity equal to the false-positive rate.

## Results

### Patient characteristics and "gold-standard" results

**Figure 2:**
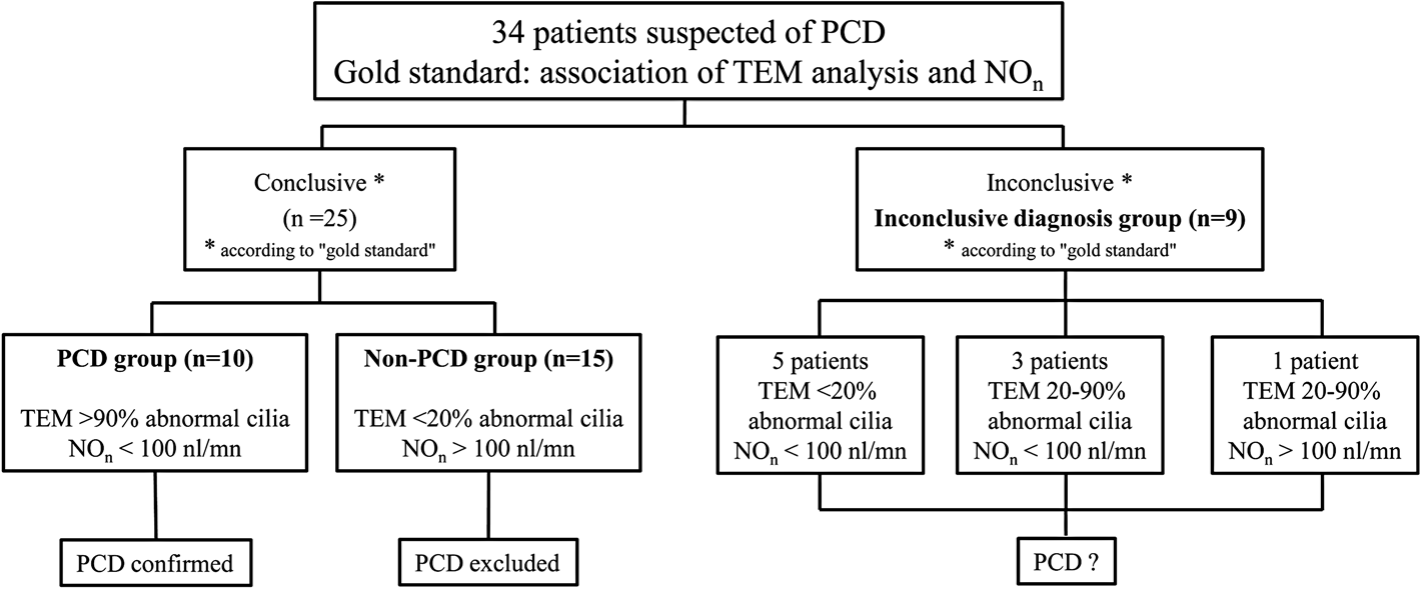
Determination of the patient groups according to the “gold standard”.

All patients exhibited diffuse bronchiectasis on HRCT scan. Two patients (# 15 and 29) had a history of smoking (2 and 10 pack-years, respectively) and had ceased smoking for more than 5 years. Patient characteristics are shown in Table 1. Results of NO_n_ measurement and TEM analysis of ciliary ultrastructure are shown in Table 2. As summarized in Figure 2, the 34 patients were divided into three groups: in the PCD group (10 patients), a diagnosis of PCD was confirmed with absence of dynein arms affecting most of the cilia combined with a low level of NO_n_; in the non-PCD group (15 patients), ciliary ultrastructure and NO_n_ measurements were considered normal, therefore excluding a diagnosis of PCD; in the inconclusive diagnosis group (9 patients), no conclusion could be reached because of discordant results between TEM and NO_n_ (Figure 2). Among these 9 inconclusive patients, 8 patients presented more than 70% normal cilia associated with abnormal NO_n_ (< 100 nl/min). The abnormal NO_n_ level was always confirmed by a second NO_n_ measurement performed at least six months later. In one patient (#33), the rate of normal cilia was below 50%, but NO_n_ was within the normal range. This patient had a second nasal biopsy and another NO_n_ measurement. The new results (50% abnormal cilia and 250 nl/min) did not modify this patient’s diagnostic status. No significant difference was observed between the three groups for age, FEV_1_, FEV_1_/VC and PaO_2_.

**Table 1:** Characteristics of non-PCD patients, PCD patients and patients with inconclusive diagnosis

**Patients (gender)**	**Age (years)**	**Onset of symptoms**	**Consanguinity**	**SI**	**Infertility**	**Respiratory status**		**FEV**_**1**_	**FEV**_**1**_**/FVC**	**PaO**_**2**_
						**Pulmonary**	**ENT**			
**Group: PCD**										
1(F)	16	Birth	Y	N	Nd	CB	SOM RS	68%	61%	95
2(F)	22	Childhood	Y	N	N	CB	SOM RS	52%	80%	99
3(M)	39	Childhood	N	N	Y	CB	SOM RS NP	80%	77%	70
4(M)	18	Childhood	N	Y	Nd	CB	RS	74%	66%	70
5(M)	10	Childhood	N	Y	Nd	CB	SOM RS	111%	87%	99
6(M)	29	Childhood	Y	Y	Y	CB	SOM RS	100%	79%	99
7(M)	28	Childhood	N	N	Nd	CB	SOM RS	90%	75%	95
8(M)	47	Childhood	Y	Y	Y	CB	SOM RS	102%	78%	83
9(F)	46	Birth	Y	Y	Nd	CB	SOM RS	100%	75%	96
10(F)	33	Childhood	Y	N	Nd	CB	RS	98%	93%	99
**Group: Non-PCD**										
11(F)	26	Childhood	N	N	Nd	CB	Normal	69%	70%	78
12(F)	27	Childhood	N	N	Y	CB	SOM RS	83%	72%	96
13(M)	41	Childhood	N	N	Nd	CB	SOM RS	86%	75%	95
14(M)	32	Birth	N	N	Nd	CB	SOM RS	50%	85%	77
15(F)	20	Adult	N	N	Nd	CB	Normal	79%	90%	100
16(F)	24	Childhood	N	N	Nd	CB	Normal	81%	87%	104
17(F)	73	Childhood	N	N	Y	CB	SOM	100%	69%	81
18(F)	40	Adult	N	N	N	CB	RS	100%	88%	94
19(F)	19	Childhood	N	N	Nd	CB	SOM	80%	73%	98
20(F)	30	Birth	N	N	N	CB	SOM	57%	77%	86
21(M)	72	Childhood	N	N	N	CB	RS	71%	47%	98
22(F)	14	Childhood	N	N	Nd	CB	Normal	81%	78%	96
23(M)	62	Childhood	N	N	N	CB	RS	63%	55%	89
24(M)	17	Childhood	N	N	Nd	CB	RS	78%	68%	100
25(M)	14	Birth	N	N	Nd	CB	SOM	82%	66%	92
**Group: Inconclusive diagnosis**										
26(F)	28	Childhood	N	N	Nd	CB	SOM RS	80%	79%	98
27(M)	26	Childhood	Y	Y	Nd	CB	SOM RS	89%	86%	85
28(F)	31	Childhood	N	N	Nd	CB	SOM RS	50%	66%	77
29(M)	59	Adult	N	N	Y	CB	RS	86%	69%	96
30(M)	44	Childhood	N	N	Y	CB	SOM RS	81%	78%	107
31(F)	26	Childhood	N	N	Nd	CB	SOM RS	41%	66%	68
32(F)	34	Childhood	Y	N	Y	CB	RS	31%	44%	64
33(F)	36	Childhood	Y	N	Y	CB	SOM RS	70%	92%	93
34(M)	21	Birth	N	N	Nd	CB	SOM RS	79%	83%	90

Abbreviations: *PCD*, primary ciliary dyskinesia; *F*, female; *M*, male; *N*, no; *Y*, yes; *SI*, situs inversus; *CB*, chronic bronchitis; *ENT*, ear nose and throat; *SOM*, serous otitis media; *RS*, rhinosinusitis; Nd: not determined; *FEV*_*1*_, forced expiratory volume in 1 second and *FVC*, forced vital capacity, PaO_2_ partial pressure of oxygen.

**Table 2:** Results of beat pattern: qualitative evaluation and quantitative analysis in non-PCD patients, PCD patients and patients with inconclusive diagnosis

**Patients**	**Qualitative evaluation**					**Quantitative analysis**		**NO**_**n**_**(nl/min)**	**TEM analysis of cilia**		
	**Abnormal beating (%)**				**Ciliary dyskinesia score**	**Weighted distance traveled per sec(μm)**	**Weighted area swept per sec (μm**^**2**^**)**		**Abnormal cilia (%)**	**Main ultrastructural defect**	**Cilia orientation**
	**Total**	**Vi**	**St**	**Ci**							
**Group: PCD**											
1	85	55	30	0	2.40	1.2	3.3	15	100	IDA+NL	Poor
2	80	70	10	0	2.35	0.5	1.3	0	100	ODA	N
3	95	90	5	0	2.83	0.0	0.0	5.9	100	ODA	N
4	50	35	15	0	1.45	6.2	17.5	5.7	92	ODA	N
5	55	5	50	0	1.4	5.8	16.0	34	100	IDA+NL	N
6	75	30	45	0	2.03	5.2	12.9	4.2	100	ODA	N
7	10	5	5	0	0.25	27.1	87.9	59.8	100	ODA	N
8	100	90	10	0	2.95	0.6	1.8	11.1	100	ODA	N
9	100	100	0	0	3	0.0	0.0	6	100	ODA	N
10	100	100	0	0	3	0.0	0.0	13	100	ODA	N
**Group: Non-PCD**											
11	0	0	0	0	0	53.3	184.6	809	7	No	N
12	10	0	10	0	0.25	82.8	267.8	260	1.5	No	N
13	5	0	5	0	0.15	53.7	148.2	219	2	No	N
14	5	0	5	0	0.15	77	256.6	194	9	No	N
15	0	0	0	0	0	76.1	236.2	661	6	No	N
16	10	0	10	0	0.25	80.9	248.8	183	2	No	N
17	20	0	20	0	0.5	75.6	251.5	100	7	No	N
18	20	0	20	0	0.5	34.1	95.8	639	3	No	N
19	25	0	25	0	0.63	67.7	218.7	282	2	No	N
20	25	20	5	0	0.73	68.1	199.8	784	3	No	N
21	20	0	20	0	0.5	39.9	116.8	264	15	CC	N
22	5	0	5	0	0.13	50.3	163.4	132	4	No	N
23	25	0	25	0	0.63	43.2	119.8	250	0	No	N
24	0	0	0	0	0	46.0	181.2	263	3	No	N
25	5	0	5	0	0.13	59.6	160.8	324	0	No	N
**Group: inconclusive diagnosis**											
26	0	0	0	0	0	73.3	214.8	83	2	No	N
27	90	80	10	0	2.65	0.0	0.0	17	6.5	Heterogeneous	N
28	60	45	15	0	1.75	9.1	27.3	15	14	Heterogeneous	Poor
29	20	0	20	0	0.5	30.3	85.6	93	21	ODA	N
30	90	90	0	0	2.7	0.0	0.0	5	30	Heterogeneous	N
31	70	0	70	0	2.1	29.3	97.9	1	0	No	N
32	55	5	45	5	1.40	28.6	90.1	1	10	Heterogeneous	Poor
33	0	0	0	0	0.0	76.0	242.8	262	55	ODA+IDA	Poor
34	60	15	40	5	1.55	5.7	17.8	6.4	22	CC	N

Abbreviations: *Vi*, Virtually immotile; *St*, Stiff; *Ci*, Circular; *NO*_*n*_, nasal nitric oxide; *TEM*, transmission electron microscopy; *PCD*, primary ciliary dyskinesia; *IDA*, inner dynein arms; *NL*, nexin links; *ODA*, outer dynein arms; *CC*, central complex; *N*, normal.

### Qualitative evaluation of ciliary beat pattern

In the PCD group, seven patients exhibited abnormal ciliary beat pattern mainly virtually immotile (Table 2). Two patients (#4 and 5) had ambiguous results with almost half of the cilia showing a normal beat pattern and a ciliary dyskinesia score of about 1.4. Finally, in the PCD group, one patient (#7) had 90% of cilia with a normal beat pattern and a ciliary dyskinesia score of 0.25.

In the non-PCD group, all patients presented at least 75% of cilia with normal beat pattern with a ciliary dyskinesia score < 0.75 (Table 2).

In the inconclusive group, patients presented very heterogeneous results with a percentage of normal beat patterns ranging from 8 to 100% and a ciliary dyskinesia score ranging from 0 to 2.7 (Table 2).

### Quantitative analysis of the ciliary beat pattern

**Figure 3:**
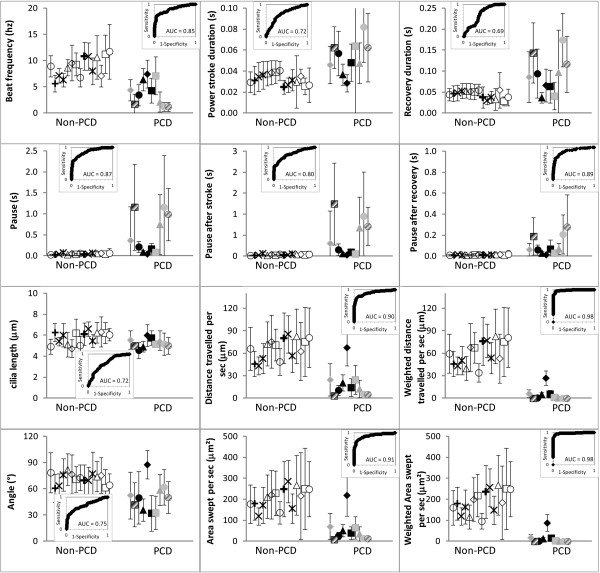
**Comparison of digital high-speed videomicroscopy parameters in the non-PCD and PCD groups.** The area under the curve (AUC) of the ROC curve was ≥ 0.69 for each parameter. The distance travelled per second (by the cilium tip) and the area swept per second (by the cilium), weighted by the percentage of beating ciliated edges, presented the best AUC (0.984 and 0.977, respectively).

**Figure 4:**
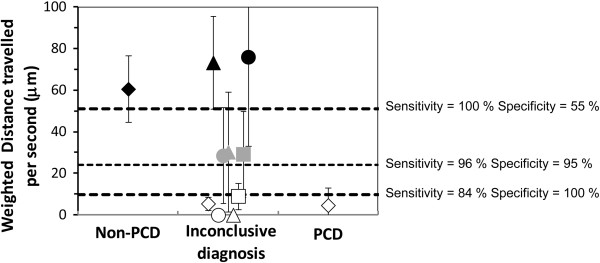
**Evaluation of the efficiency of digital high-speed videomicroscopy in nine patients with an inconclusive diagnosis.** In this group, the mean distance travelled by the cilium tip per second weighted by the percentage of beating ciliated edges, was used to discriminate patients. The PCD and non-PCD cutoffs were < 10 μm and > 51 μm respectively. The symbols of the non-PCD and PCD groups represent the mean values ± standard deviation. In the inconclusive group, white symbols represent patients in whom PCD was possibly confirmed, black symbols represent patients in whom PCD was possibly excluded and grey symbols represent patients in whom digital high-speed videomicroscopy was not helpful for PCD diagnosis.

All digital high-speed videomicroscopy parameters were able to be determined in the 34 patients. In the 25 conclusive patients, all digital high-speed videomicroscopy parameters tested were significantly different between the PCD and non-PCD groups. All measurements (n=250) obtained in these two groups were used to test the sensitivity and specificity of each digital high-speed videomicroscopy parameter for PCD status. The AUC of the ROC curve was ≥ 0.69 for each parameter (Figure 3). The weighted distance traveled by the cilium per second and the weighted area swept per second by the cilium had the best AUC (0.984 and 0.977, respectively). For the weighted distance travelled per second, a cutoff of 24 μm gave a sensitivity and specificity of 96% and 95%, respectively (Figure 4). The cutoff values of 51 μm and 10 μm gave 100% sensitivity (55% specificity) and 100% specificity (84% sensitivity), respectively. For the weighted area swept per second, a cutoff of 62 μm^2^ gave a sensitivity and specificity of 94% and 94%, respectively. The cutoff values of 221 μm^2^ and 22 μm^2^ gave 100% sensitivity (27% specificity) and 100% specificity (83% sensitivity), respectively.

In the PCD group, the beat frequency tended to be lower in patients with outer dynein arm (ODA) defects and the beating angle tended to be lower in patients with inner dynein arm (IDA) defects. However, the small number of patients did not allow any statistical comparisons.

In the inconclusive diagnosis group, the quantitative parameters provided by digital high-speed videomicroscopy were helpful to orientate the diagnosis of PCD in 6/9 patients: in four patients, mean weighted distance traveled per second was below the 100% specificity cutoff, supporting the diagnosis of PCD and for two patients, this parameter was above the 100% sensitivity cutoff, probably excluding the diagnosis of PCD. For the remaining 3/9 patients of the inconclusive diagnosis group, the mean weighted distance traveled per second was situated between these two cutoff values and no conclusion could be reached (Figure 4).

## Discussion

This study shows that digital high-speed videomicroscopy can be used to precisely analyze ciliary beat pattern and can be helpful for PCD diagnosis. Quantitative parameters that are useful to distinguish between PCD and non-PCD patients were defined. Among these parameters, the weighted distance travelled per second provided the best sensitivity and specificity.

The set of results of ciliary ultrastructure and NO_n_ measurement used as “gold standard” for PCD diagnosis clearly distinguished between the PCD and non-PCD groups. TEM analysis of cilia is generally considered as the reference method for the diagnosis of PCD [1,7,21,22]. NO_n_ measurement, which is now classically used in diagnostic procedures [8,9,11,21,23], was combined with TEM analysis in order to more accurately define the PCD and non-PCD groups. However, the variants of PCD with normal ciliary ultrastructure are clearly a limitation of such a gold standard as TEM alone misses the diagnosis. Nevertheless, the association of TEM with NO_n_ measurement in our study allowed to de-emphasize this limitation as the patients with normal ciliary ultrastructure and low No_n_ were classified as inconclusive. Evaluation of ciliary beat pattern in culture has already been proposed [24] to distinguish primary and secondary ciliary dyskinesia. This procedure was not used in the present study because, as previously reported [25], cilia fail to grow in up to 46% of cultures.

Digital high-speed videomicroscopy has been developed to precisely assess ciliary beating by slow motion analysis [26] and can be used to study the movement of a cilium throughout the beat cycle. Chilvers et al. reported a slow, short and stiff flickering beat pattern in PCD patients with an isolated outer dynein arm defect and a stiff forward power stroke with markedly reduced amplitude in PCD patients with an isolated inner dynein arm defect [14]. Similarly, we found that inner arm defects (patients # 1 and 5) were associated with a relatively high rate of stiff pattern while outer dynein arm defects (patients # 2, 3, 4, 6, 7, 8, 9 and 10) were associated with a relatively high rate of virtually immotile pattern.

We hypothesized that digital high-speed videomicroscopy can be used to develop quantitative parameters that objectively characterize ciliary beat pattern. Firstly, all of the proposed quantitative parameters were able to be determined in all patients, meaning that beating cilia were always detected, even in the absence of dynein arms. In our experience, the average duration of a complete assessment (including video recording and analysis) is about one hour per patient, allowing this technique to be applied in clinical practice. The mean ciliary beat frequency measured in this study was significantly lower in PCD patients than in non-PCD patients with chronic airway infections, as reported with conventional techniques [3,10,14,26]. The beating angle can be considered to be a parameter evaluating the amplitude of the ciliary beat. Due to the flexible nature of ciliary beat, beating angle remains of course only an indicator of amplitude. In the future, the measurement of the bending angle of the cilia, especially during the power stroke, might be an interesting parameter. However, this measurement will require to increase the amount of measured points on the video of the beating cilia and to develop new algorithms of calculation. All quantitative parameters were significantly different between PCD and non-PCD groups. Weighted distance travelled per second could be useful for PCD diagnosis, as this parameter appeared to be the most discriminative with no overlapping results between PCD and non-PCD groups.

**Figure 5:**
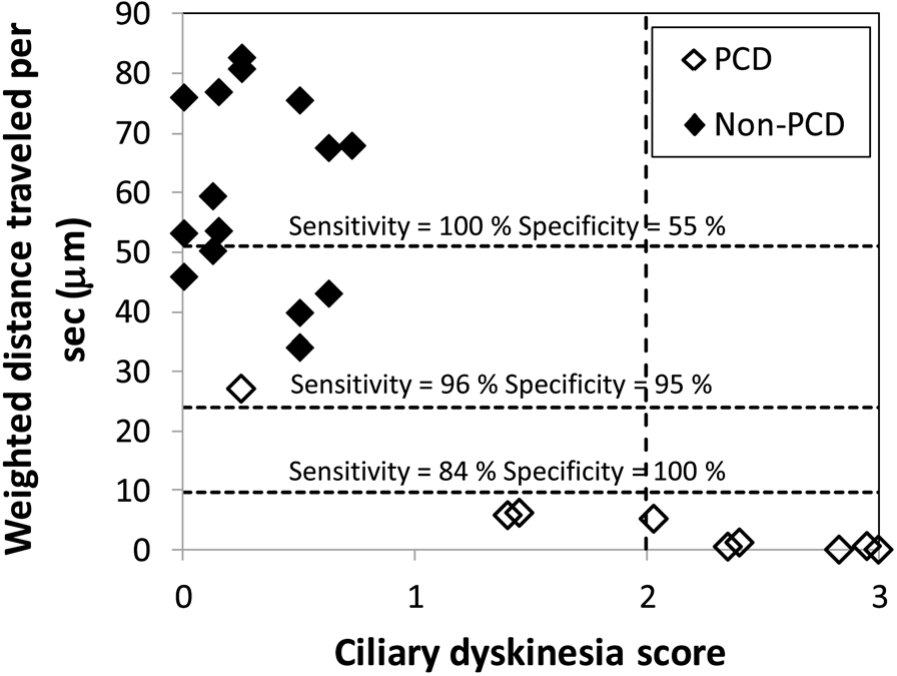
**Quantitative analysis versus qualitative evaluation.** The results of the weighted distance travelled per second is plotted versus the dyskinesia score in the patients with conclusive gold standard. According to Stannard et al. [13], a score ≥ 2 was described as the best predictor of PCD.

The results provided by qualitative evaluation and quantitative analysis were not always concordant. In the patients with a conclusive gold standard diagnosis, the two methods were always concordant when the ciliary dyskinesia score ≥ 2 or ≤ 0.15, (14 patients, see Table 2 and Figure 5), i.e. when ciliary beat pattern was virtually immotile or normal, respectively. In the cases of ciliary dyskinesia score < 2 and or > 0.15 (11 patients see Table 2 and Figure 5), i.e. when a moderate alteration of ciliary beat pattern was exhibited, three situations were observed: in 2/11 patients (# 4 and 5), the two methods were discordant and the PCD diagnosis was in accordance with the quantitative analysis; in 8/11 patients (# 12, 16, 17, 18, 19, 20, 21 and 23) the quantitative analysis concluded to the non-PCD diagnosis with a ≥ 98% sensitivity and the qualitative evaluation showed a low dyskinesia score (between 0.25 and 0.5); in 1/11 patient (# 7) with a conclusive PCD diagnosis, qualitative evaluation showed a low dyskinesia score (0.25) while the quantitative parameters were just below the threshold of 96% sensitivity and 95% specificity. It is difficult to pinpoint the reasons of the discrepancies observed between quantitative and qualitative methods, as the two methods are based on the same concept, i.e., describing a movement from the same high speed video recording. We can speculate that qualitative evaluation does not always allow easy assessment of beat pattern. Scoring ciliary dyskinesia from 1 to 3 depending on the extent of abnormal beating along the edge, as proposed by Chilvers et al. [20], is clearly more subjective than a physical measurement in a continuous space. Our results suggest that the proposed quantitative analysis is more precise and more sensitive than qualitative evaluation. This gain of precision is especially useful when cilia are still beating but with moderate alterations (more than 40% of patients referred for suspicion of PCD in our center).

In more than one quarter of the patients, gold standard was inconclusive. This high rate probably can be explained by the extensive genetic heterogeneity of PCD. Such a result demonstrates the need to have a new method allowing to better defining PCD. The proposed method is a trial to get a better classification of PCD, based on the dyskinectic pattern. In the inconclusive group, the proposed parameters can be helpful to assess the diagnosis of PCD while the measurement of ciliary beat frequency alone is not helpful. The weighted distance travelled per second is the most discriminating parameter for this purpose. Use of this type of procedure could reclassify two patients as possibly excluded PCD (patients # 26 and 33) and four patients as possibly confirmed PCD (patients # 27, 28, 30 and 34). Very interestingly, for one of these patients (#34), the diagnosis of PCD was confirmed by genetic analysis showing *RSPH9* mutations (data not shown). Nevertheless, three patients remained inconclusive for PCD status (patients # 29, 31, 32; see grey symbols in Figure 4). Molecular genetics studies would greatly improve diagnostic efficiency, but gene mutations have been reported in fewer than 50% of PCD patients to date [3,22].

## Conclusions

In conclusion, qualitative evaluation remains the first-line method to study ciliary beating and is sufficient when the beat pattern is normal or virtually immotile. However, quantitative analysis provides more precise and more sensitive results in the presence of moderate alterations (observed in almost one half of patients in this series). Therefore, it could be very useful in PCD patients representing a challenge for diagnosis, as those with Kartagener syndrome and no ultrastructural defect (patient # 27) [7] and those with central complex defect and low percentage of abnormal cilia combined with residual beating cilia (patient # 34) [16,27,28]. Quantitative analysis of beat pattern can also be proposed to select patients eligible for TEM by using a cutoff for weighted distance travelled per second ensuring 100% sensitivity (even with a specificity of 55%). This type of selection would decrease by almost one third the indications for TEM analysis in patients with suspected PCD.

This pilot study should promote additional studies on a larger series of patients. Quantitative approaches of ciliary motility are potentially useful to define dyskinesia and dyskinetic pattern for the diagnosis of PCD.

## Abbreviations

AUC: Area under curve; HRCT: High-resolution chest computed tomography; IDA: Inner dynein arms; NO_n_: Nasal nitric oxide; ODA: Outer dynein arms; PCD: Primary ciliary dyskinesia; TEM: Transmission electron microscopy.

## Competing interests

The authors declare that they have no competing interests.

## Authors' contributions

JFP participated in study design and writing of the paper and carried out the nasal sampling and high-speed videomicroscopy recording and analysis. LB participated in the design and the writing of the paper. GCP participated in study design and data acquisition. FZL participated in study design, carried out nasal NO measurements and performed statistical analysis. AMV provided technical assistance for TEM analysis. SB participated in study design and data acquisition. BC participated in study design and writing of the paper. SA participated in study design and writing of the paper and carried out molecular genetic studies. AC participated in study design and writing of the paper and carried out nasal sampling. BH participated in study design and writing of the paper. EE participated in study design and writing of the paper and carried out TEM analysis. BL participated in study design and writing of the paper and carried out high-speed videomicroscopy recording and analysis. All authors read and approved the final manuscript.

## Supplementary Material

Additional file 1 Example of a high-speed videomicroscopy recording obtained from a patient with dyskinetic cilia. Click here for file
